# Neonatal Renal Failure After Maternal Motor Vehicle Accident

**DOI:** 10.3390/children12091179

**Published:** 2025-09-04

**Authors:** Jasmine Y. Massoumi, Caroline M. Bebawy, Sheema Gaffar

**Affiliations:** 1 Department of Pediatrics, Division of Neonatology, Los Angeles General Medical Center, Los Angeles, CA 90033, USA; 2 Department of Pediatrics, Children’s Hospital of Los Angeles, Los Angeles, CA 90027, USA; carolinembebawy@gmail.com; 3 Fetal and Neonatal Institute, Division of Neonatology, Children’s Hospital of Los Angeles, Los Angeles, CA 90027, USA; sgaffar@chla.usc.edu

**Keywords:** neonatal renal failure, preterm neonate, motor vehicle accident

## Abstract

Background: Motor vehicle accidents account for the majority of abdominal trauma in pregnancy and can result in fetal morbidity and mortality. With advancing gestation, the fetus becomes more vulnerable to injury. Case presentation: A preterm neonate is born at 32 weeks’ gestation via cesarean section due to placental abruption after maternal motor vehicle accident. Initially, the infant presented with anemia, thrombocytopenia, and acute kidney injury in the setting of renal contusions. Results: Hyponatremia, acidosis, oliguria, and uremia progressed to frank anuric renal failure, requiring several months of hemodialysis before transition to peritoneal dialysis for chronic renal replacement therapy at home. Conclusions: Fetal renal injury resulting in postnatal renal failure is a rare but potentially devastating complication of blunt abdominal injury during pregnancy. Sonographic and laboratory evaluation of a neonate with suspected in utero injury after maternal motor vehicle accident is imperative, as is a high index of suspicion for neonatal renal injury.

## 1. Introduction

Motor vehicle accident (MVA) accounts for most traumatic injury in pregnancy [1]. Although as much as 90% of traumatic maternal injuries in pregnancy are classified as ‘minor’, fetal injury may occur in 60–70% of these cases [2,3,4]. Predictors of fetal morbidity and mortality include direct uterine trauma, uterine rupture, placental abruption, prolonged maternal hypotension or hypoxemia, and maternal death [1,3,5]. Although the uterus and amniotic fluid function as cushions for the fetus, the protection afforded is limited [6], especially with advancing gestation when maternal and placental anatomy changes. As the uterus expands, it exceeds the capacity of the pelvis and becomes an intra-abdominal organ. Without the protection of the bony pelvis, both uterine and fetal vulnerability to blunt injury increases [2,3,5]. Early in pregnancy, direct fetal injury is rare, as the size of the fetus and uterus are comparable. Direct fetal injury occurs more commonly in late pregnancy, when the fetus is larger than the uterus and more vulnerable to blunt and penetrating forces. Intracranial and skeletal injury are most common due to fetal head position and acceleration–deceleration forces [1,5,6,7]. Indirect fetal injury can occur from any force that compromises placental blood flow, such as shearing, compression, or embolism [8].

Fetal nephrogenesis begins at 5 weeks of gestation and continues until 34–36 weeks’ gestation [9,10]. With premature birth, there is incomplete nephron development and decreased nephron number, translating to increased risk of neonatal acute kidney injury (AKI) and subsequent chronic kidney disease later in childhood [9,10]. As such, risk factors for neonatal AKI and acute renal failure in infants include prematurity, hypotension, hypoxemia, thrombocytopenia, coagulopathy, and nephrotoxin exposure [10,11]. Furthermore, adult kidneys are protected by the ribcage, while infant kidneys are relatively large and positioned lower in the abdomen, rendering them unprotected [12,13]. Infants and children have a thinner perinephric fat layer than adults, which is not only less protective but also less secure, with only the vasculature and the ureter anchoring each kidney in place [13]. Together, these anatomic and developmental factors make the developing kidney vulnerable to serious injury with trauma. Contusion, avulsion, and hypoxia-ischemia are common mechanisms of injury to the kidneys [13]. With renal contusions, most parenchymal injury is localized to the upper and lower poles of the kidney, causing them to appear hyperechogenic on ultrasound secondary to edema, with additional focal echogenicity at areas of hematoma formation. Renovascular avulsion typically presents immediately with unilateral parenchymal hemorrhage and later with segmental ischemia, depending on the degree of trauma at the renal hilum. Hypoxia-ischemia may affect entire vascular territories, potentially presenting as a wedge-shaped echogenicity in the renal cortex or as diffuse cortical echogenicity [13].

We describe a normal preterm neonate who sustained severe renal contusions and renal cortical necrosis from maternal MVA and placental abruption at 32 weeks’ gestation, resulting in oliguric renal failure. The neonate was cared for in a level 3 neonatal intensive care unit (NICU) at the birth hospital (Hospital 1, the community hospital where the mother received emergency care after MVA) for the first 4 days of life until refractory renal failure became evident, necessitating transfer to a level 4 NICU (Hospital 2, a large children’s hospital and regional referral center) for renal replacement therapy [14].

## 2. Case

The mother was the front-seat passenger of a vehicle involved in a head-on MVA. Direct impact occurred on the driver’s side, on a surface-street, at approximately 20–30 miles per hour, with airbag deployment. The mother was restrained with a shoulder and lap belt and not ejected, but did lose consciousness on impact. Immediately after the collision, emergency medical services arrived, extricated her from the vehicle, and transported her to the nearest hospital (Hospital 1), where she regained consciousness one hour later.

In the Emergency Department of Hospital 1, the mother presented with leg pain, vaginal bleeding, and pregnancy at 32 weeks. She denied abdominal pain but reported decreased fetal movement compared to earlier in the day. She was an otherwise healthy 36-year-old gravida 2 para 1, with good prenatal care. Maternal serologies were negative except for unknown Group B *Streptococcus* status. She was diagnosed with a right ankle fracture. The remainder of the primary trauma survey was unremarkable. However, evaluation by the obstetrician in the context of possible placental abruption revealed non-reassuring fetal heart tracings. Thus, the mother was taken to the obstetrics unit for emergent cesarean section.

A female infant was born at Hospital 1, weighing 1.775 kg (51st percentile) and was pale, hypotonic, and with no respiratory effort, requiring immediate endotracheal intubation. APGAR scores were 2, 4, and 6 at 1, 5, and 10 min, respectively. The neonate was admitted to the NICU for further management of respiratory failure, prematurity, and trauma. Cord gases were not able to be obtained for unclear reasons. On admission, the patient’s vital signs were within normal limits except for one transiently low cuff blood pressure that improved after red blood cell transfusion. At one hour of life, arterial blood gas from the neonate resulted with pH 7.27, PaCO_2_ 41 mmHg, PaO_2_ 40 mmHg, HCO_3_ 19 mEq/L, and base deficit 7.6.

In the level 3 NICU of Hospital 1, the infant was started on ampicillin and gentamicin in the setting of maternal fever and no intrapartum antibiotics. Access was obtained via peripherally inserted central venous catheter located in the mid-superior vena cava and umbilical arterial catheter located at the 6th thoracic vertebral level. The infant was initially started on dextrose- and calcium-containing intravenous fluids at 80 mL/kg per day and transitioned to intravenous hyperalimentation with protein (2.3 g/kg) and lipids at 100 mL/kg per day on day of life (DOL) 2. There was also anemia with hemoglobin level 10.7 g/L, requiring 20 mL/kg red blood cell transfusion on DOL 0, and moderate thrombocytopenia with platelet count 46 × 10^9^/L, requiring 15 mL/kg platelet transfusion on DOL 3. The patient received two doses of surfactant for respiratory distress syndrome, enabling transition on DOL 3 from pressure-control ventilation with 25 cm H_2_O maximum inspiratory pressure, 5 cm H_2_O expiratory pressure, and 0.30 O_2_ concentration to 2 L per minute high-flow nasal cannula with 0.21 to 0.25 O_2_ concentration.

Although blood culture drawn at birth had no growth, the respiratory failure, hypotension, edema, oliguria, and overall clinical instability of the infant in the context of maternal fever raised clinical concern for early-onset sepsis, necessitating treatment with 5 days of antibiotics, initially with an aminoglycoside (gentamicin) and a broad-spectrum penicillin (ampicillin). When AKI was evident on DOL 3, the nephrotoxic aminoglycoside (gentamicin) was discontinued and a third-generation cephalosporin (ceftazidime) was started to complete the course. Cranial ultrasound from Hospital 1 showed right-side grade 1 germinal matrix hemorrhage. The patient became fluid overloaded, requiring a decrease in total fluids to 80 mL/kg per day and remained restricted to this volume until transfer on DOL 4. Abdominal ultrasound on DOL 1 demonstrated trace free fluid in all four quadrants with no comment on the structure or size of the kidneys. As the oliguria progressed to anuria, diuretic challenge with furosemide was performed, first with intermittent doses of 1 mg/kg every 4 h, followed by a continuous infusion of 0.5 mg/kg per hour. The urine output did not improve, so low-dose dopamine infusion was added at 2 mcg/kg per minute. Despite escalating interventions for 24 h, the infant remained in stage 3 renal failure (per Kidney Disease Improving Global Outcomes neonatal nomenclature [15]) (Table 1), prompting transfer on DOL 4 to a level 4 NICU for pediatric nephrology evaluation and neonatal renal replacement therapy.

On arrival at the quaternary NICU at Hospital 2 on DOL 4, the infant was hypertensive and 0.5 kg above birthweight. Arterial blood pressure on admission was 97/69 mmHg with mean 82 mmHg. Physical examination was notable for generalized edema, mild hypotonia with weak grasp reflex, weak and incomplete Moro reflex, and normal suck reflex. Admission labs were significant for hyponatremia (130 mmol/L), metabolic acidosis (bicarbonate 17 mmol/L, lactate level 1.6 mmol/L), uremia (20.7 mmol/L), and elevated serum creatinine (342 µmol/L). Urinalysis showed proteinuria (1 g/L) and both macroscopic and microscopic hematuria. Hemoglobin level (15.5 g/L) and platelet count (250 × 10^9^/L) were normal, reflecting the blood products transfused at Hospital 1.

**Figure 1:**
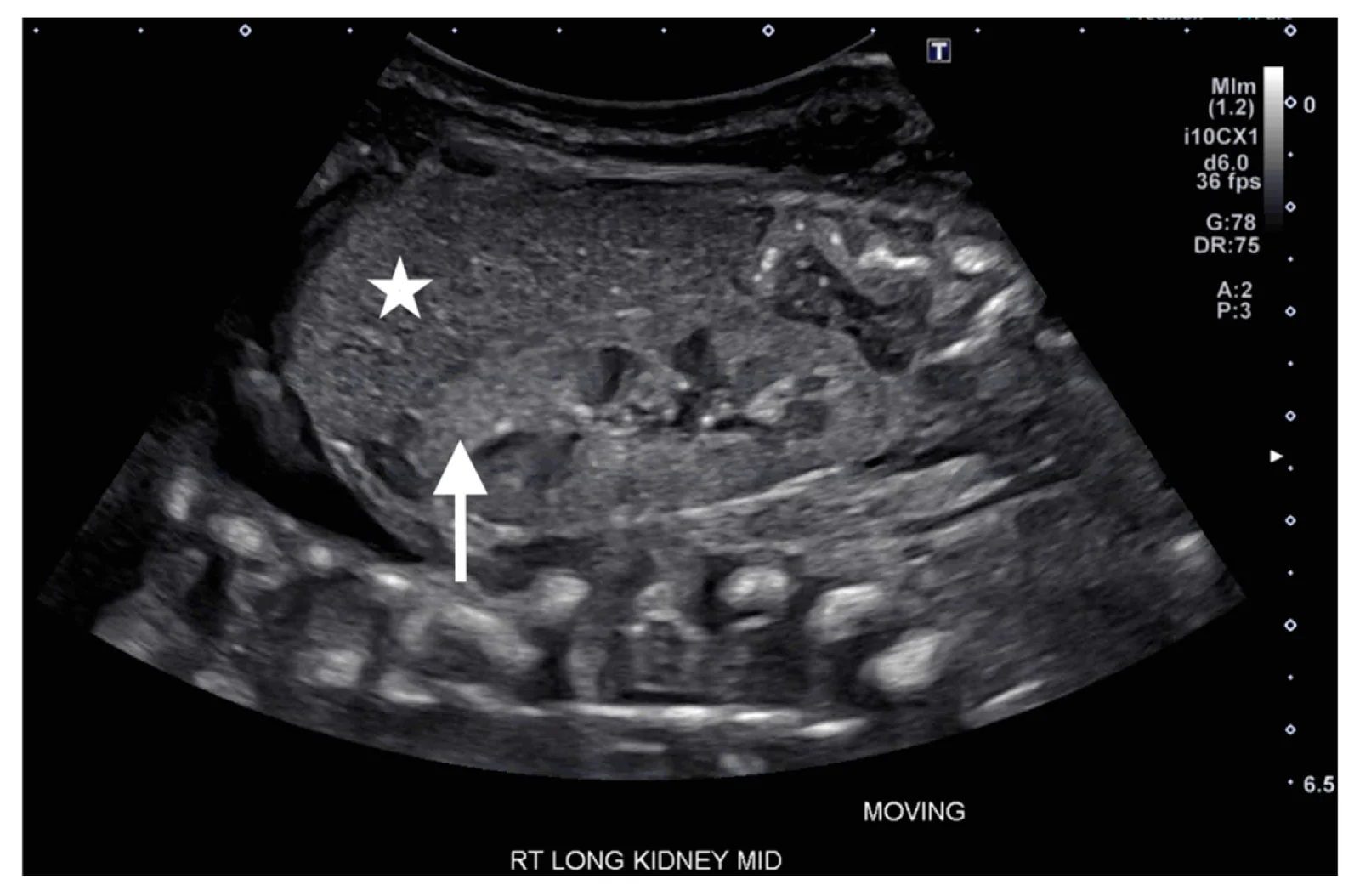
Ultrasound of right kidney on day of life 5 showing increased echogenicity (arrow) compared to that of the adjacent liver (asterisk).

**Figure 2:**
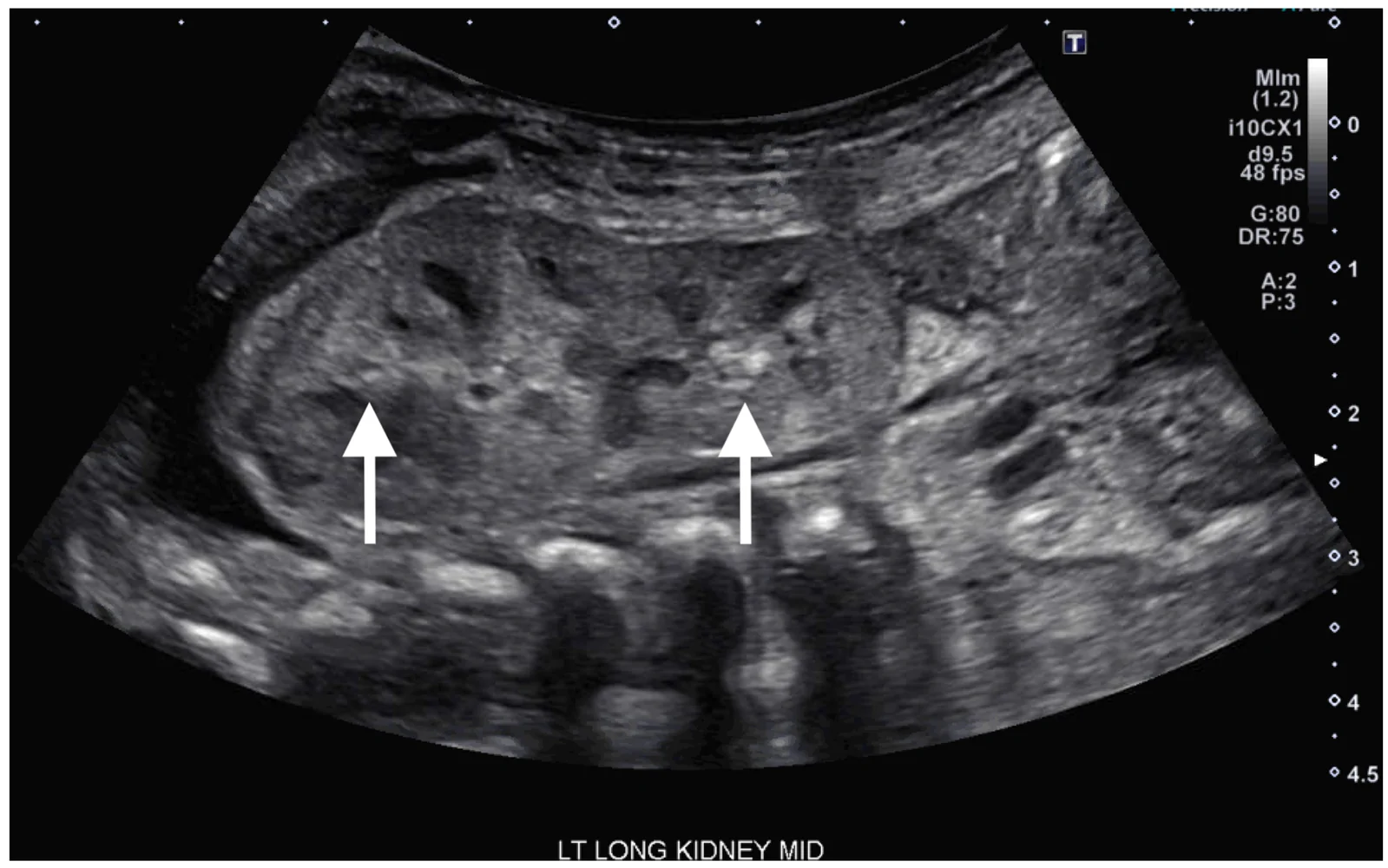
Ultrasound of left kidney on day of life 5 showing increased focal echogenicity of the upper and lower poles (arrows).

**Figure 3:**
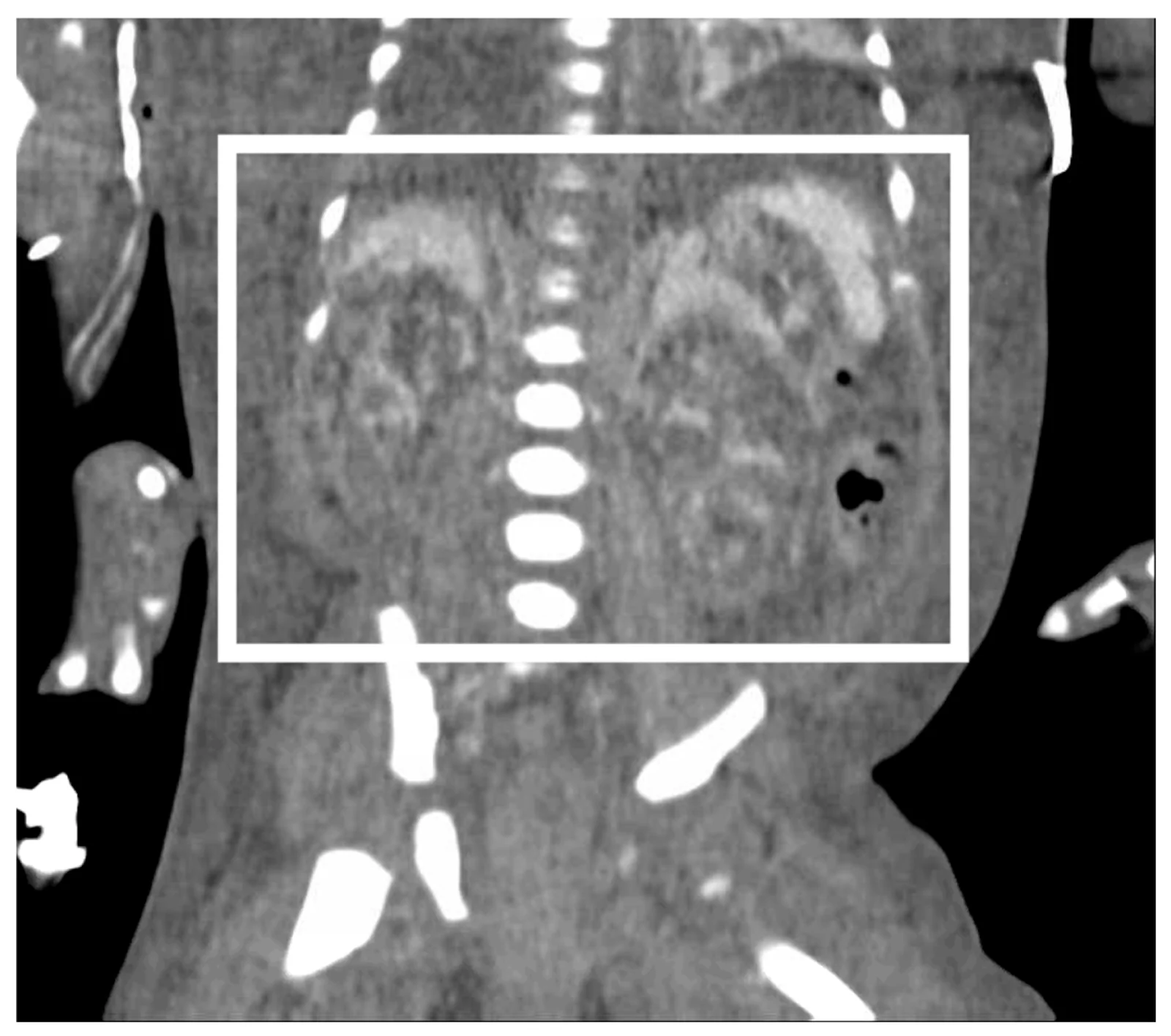
Computed tomography image utilizing intravenous contrast on day of life 5 showing bilateral patchy opacification of the external renal capsule and central renal medulla (box).

**Figure 4:**
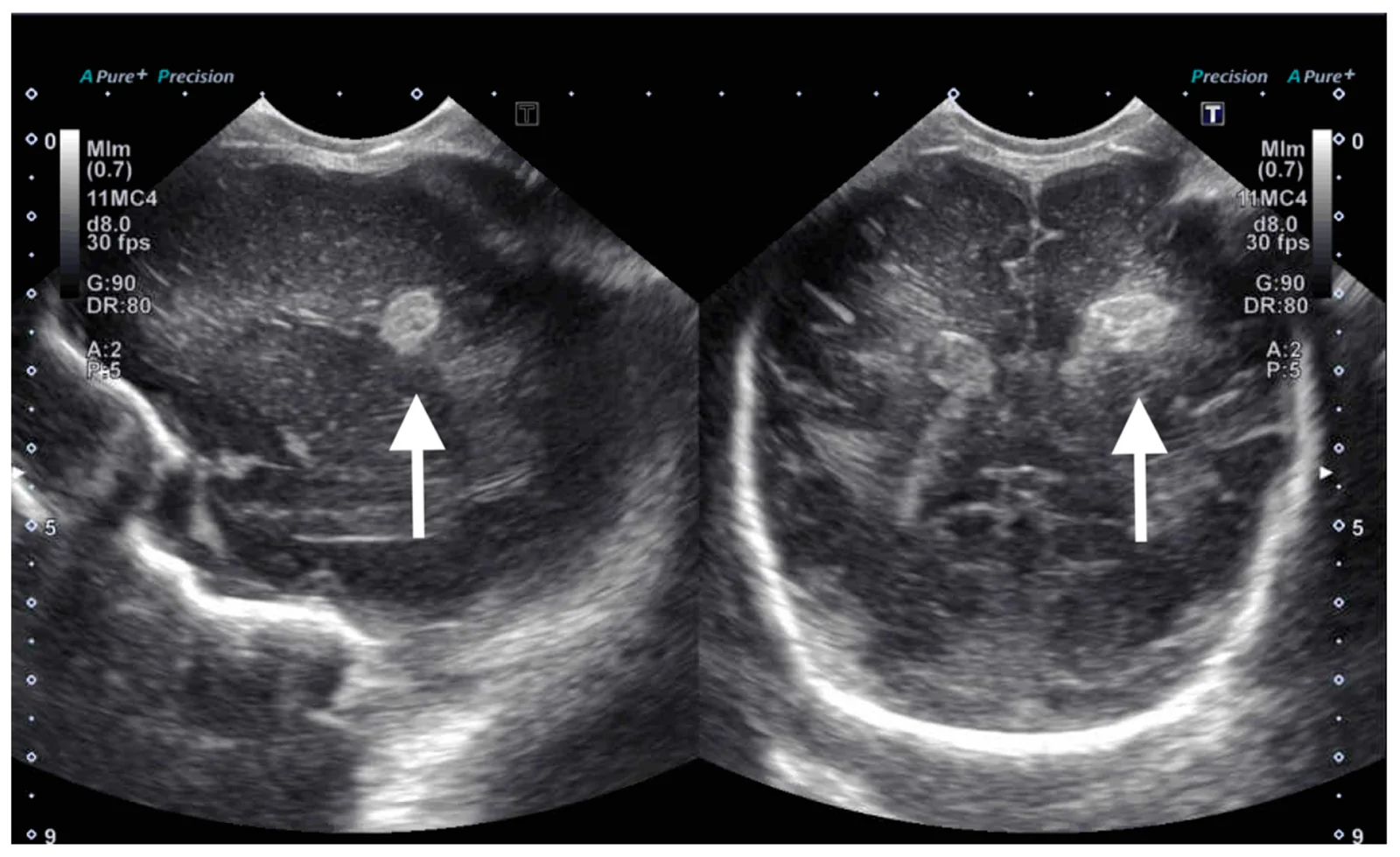
Sagittal (**left**) and coronal (**right**) views of cranial ultrasound from day of life 5 showing focal echogenicity on the left side of the posterior frontal lobe white matter (arrows) with no intraventricular hemorrhage or ventriculomegaly.

On DOL 5, the patient developed worsening abdominal distention with increased free fluid, rising lactate level, and continued oliguria with minimal brown-colored output, indicating potential bladder rupture. Renal ultrasound at that time demonstrated structurally normal kidneys and ureters, with contusive injuries in the parenchyma (Figure 1 and Figure 2). Pediatric urology was consulted. Diagnostic paracenteses and non-voiding cystogram showed no urinary leak. Computed tomography (CT) of the abdomen and pelvis was performed to further evaluate for free fluid or perforated viscus showed no renal contusion, but noted both kidneys had patchy opacification in the capsule and medulla, consistent with bilateral renal cortical necrosis (Figure 3). Cranial ultrasound was also performed at this time and showed focal parenchymal hemorrhage in the posterior portion of the left frontal lobe (Figure 4).

From DOLs 4 to 6, the patient remained nil per os with fluids further restricted to 60 mL/kg per day to manage fluid overload and hyponatremia. Once the electrolytes began to improve, the dextrose-containing intravenous fluids were transitioned to intravenous hyperalimentation with lipids.

By DOL 6, the decision was made to initiate continuous veno-venous hemodialysis for fluid overload and renal failure. On DOL 7, a single lumen 6 French hemodialysis catheter was placed into the right internal jugular vein, and dialysis was initiated on DOL 8 with the Cardio-Renal Pediatric Dialysis Emergency Machine (Carpediem™, Medtronic, Minneapolis, MN, USA). The goal on the first day of dialysis was to decrease fluid overload by 25%, translating to 250 mL removed over 20 h. Citrate was used for anticoagulation instead of heparin due to the infant’s intracranial hemorrhage. The patient tolerated this treatment overall, with some expected blood pressure lability but without significant bleeding, so this dialysis regimen was continued daily.

Hemodynamic instability precluded initiation of feeds until DOL 24. As the dialysis regimen was adjusted and optimized, intermittent hypotension necessitated dopamine infusion (maximum 11 mcg/kg/min) for vasopressor support. Even after initiation of enteral feeds, the hypotension during dialysis often warranted pausing feeds in order to prioritize cardiorespiratory stabilization and evaluate for other potential causes of hemodynamic perturbations such as sepsis.

Noncontrast brain MRI obtained on DOL 41, at term corrected gestation, demonstrated no intraventricular hemorrhage and resolving multicompartment hemorrhage, consistent with coup–contrecoup shearing injury. There was no cerebral edema, loss of gray-white cortical differentiation, nor injury to any deep structures to suggest hypoxia-ischemia.

The patient eventually required gastrostomy tube placement, which was coordinated with peritoneal dialysis catheter placement. The infant was reintubated for this operation on DOL 64 and was extubated on DOL 84 to non-invasive positive pressure ventilation. Hemodialysis was continued at this time to allow the peritoneal dialysis catheter tract to heal and mature. The infant experienced intermittent hypertension (systolic blood pressure greater than 100 mmHg) due to fluid overload, which was treated with nicardipine infusion and more fluid removal. Peritoneal dialysis was initiated on DOL 83 with low fill volumes and prolonged duration of 20 h to ensure tolerance. She did well, and was gradually advanced to full fill volumes and 8 h treatment duration that ran overnight. She was also weaned off respiratory support entirely by DOL 98, reached full gastrostomy tube feeds by DOL 105, and was discharged home on DOL 140 with low-phosphorus formula and a pediatric renal multivitamin. The infant continues to undergo nightly peritoneal dialysis at home and sees pediatric nephrology, occupational therapy, and high-risk neurodevelopment clinic as an outpatient.

## 3. Discussion

We present a unique case of fetal renal injury after maternal MVA and placental abruption at 32 weeks resulting in neonatal renal failure. This was likely multifactorial, involving contusive/crush injury, decreased nephrogenesis with underdeveloped kidneys at 32 weeks’ gestation, and possible hypoxic injury with placental abruption and early neonatal anemia, culminating in necrosis of the renal cortex and medulla and clinically manifest as oliguria. The fetus also sustained focal intracranial hemorrhage and traumatic brain injury likely from acceleration–deceleration forces that sheared delicate blood vessels. The neonate ultimately required renal replacement therapy in the NICU, initially with hemodialysis, followed by intermittent peritoneal dialysis.

It is surprising that this infant developed such refractory renal failure since birth, especially in the setting of no obvious predisposing factors and the rarity of neonatal renal failure. Prior to MVA, the fetus was developing normally with no known intrauterine pathology. Fetal growth in the 51st percentile at the time of delivery suggests no significant placental insufficiency or fetal anemia to potentially cause diminished blood flow to the developing kidneys. Postnatal renal ultrasound corroborated this with structurally normal kidneys measuring 4.5 cm and 4.6 cm, appropriate for gestational age. However, stage 3 renal failure was evident with persistent urine output less than 0.3 mL/kg per day, serum creatinine increase from 1.27 µmol/L (DOL 1) to 3.57 µmol/L (DOL 4), hyponatremia, and fluid overload on physical examination.

In utero, the majority of fetal cardiac output is directed to the brain and only 2–6% is directed to the kidneys [16]. Acute placental abruption compromises oxygenated blood flow to all fetal organs, resulting in hypoxic injury and end-organ ischemia [6,17]. This infant was found to have bilateral renal cortical necrosis on abdominal CT performed on DOL 5, initially presumed to be the result of hypoxia-ischemia from placental abruption, but surprisingly had no signs of regional or diffuse hypoxia-ischemia on brain MRI on DOL 41. This discrepancy suggests that the renal cortical necrosis was less likely from hypoxia-ischemia, as all downstream end-organs should be affected with global hypoxia, and more likely multifactorial, from a combination of trauma, prematurity, and potentially some hypoxia. An alternative hypothesis that may explain this distribution of injury involves crushing forces from maternal organs displaced during the MVA. A severe crush injury to the fetal kidneys may result in diffuse necrosis of the renal cortex and would not affect the brain. Another possibility involves microemboli in the renal circulation and resultant diffuse ischemia to the renal cortex, although there was no wedge-shaped injury on renal ultrasound and no potential source of embolism was identified.

The American College of Obstetricians and Gynecologists recommends laboratory work-up and imaging to evaluate a pregnant mother after traumatic injury [3]. Based on the mechanism of injury and type of trauma sustained (i.e., penetrating, non-penetrating, blunt, crush, etc.), complete blood cell count, coagulation profile, serum chemistry, and type and screen may be obtained [3]. Regardless of trauma severity, obstetric ultrasound is necessary to assess for fetal wellness, evaluate amniotic fluid level (e.g., AFI), estimate gestational age, and identify signs of placental abruption [3]. If a woman presents with bleeding after trauma or MVA, a Kleihauer–Betke test can delineate and quantify fetal and maternal blood loss. Severity of maternal injury does not always correlate with the degree of fetal injury [3,6,10], necessitating a thorough work-up with any reported abdominal injury, particularly later in pregnancy. Focused abdominal sonography (i.e., F.A.S.T. exam) may be performed on the mother to rapidly screen for intra-abdominal free fluid and other traumatic injuries that may require urgent surgical intervention [3].

There are no guidelines for evaluation of the neonate after suspected in utero trauma, even though young infants and toddlers less than 4 years of age are more likely than older children and adults to be injured in MVA [18]. In older children, if there is clinical suspicion for abdominal injury, CT abdomen/pelvis or CT urogram may be considered [19]. Although CT is a poor predictor of “hollow” organ injury (i.e., bowel and mesentery), in children, it may be useful for identifying solid organ injury, including renal contusions or parenchymal injury, damage to renal vasculature, and rupture of the kidney, bladder, or ureter [19,20,21]. Urinalysis is an essential part of infant evaluation after abdominal trauma as hematuria indicates bleeding in the urinary tract and warrants further imaging with ultrasound or CT [13]. Clinical history, physical exam, and mechanism of injury can guide a clinician’s degree of suspicion for neonatal injury, but the neonatal workup after trauma should be standardized and should include a low threshold for pediatric surgery evaluation or transfer to a higher-level NICU with appropriate pediatric subspecialist availability [14].

In this premature neonate who developed severe AKI and renal failure, initial abdominal ultrasound showed renal contusions with free fluid in the abdomen, CT demonstrated no ruptured viscus with incidental renal cortical necrosis, and cystogram confirmed no urinary extravasation. Serial cranial ultrasounds permitted screening for intracranial hemorrhage prior to systemic anticoagulation for hemodialysis, with resolution of hemorrhage confirmed on brain MRI, highlighting the importance of multi-organ evaluation in a neonate presenting after trauma.

Lastly, appropriate use of passenger restraints is paramount for maternal and infant safety, as they distribute force away from the body and prevent vehicular ejection [5,22,23]. When seat belts are used improperly, specifically with the lap belt across a gravid abdomen, pressure and force can be directly transmitted to the body, increasing the risk of maternal and fetal morbidity [24,25]. Car seats and seat belts mitigate severity of injury in MVA but do not prevent injury [3]. MVA are, unfortunately, common, underscoring the importance of seat belt and car seat safety counseling from obstetricians and pediatricians at every prenatal and postnatal opportunity.

## 4. Conclusions

Fetal renal injury can occur after maternal MVA and placental abruption, resulting in neonatal renal failure. In severe cases, renal replacement therapy may be required. Postnatal evaluation of a neonate after suspected in utero trauma should include cranial ultrasound, abdominal ultrasound or focused renal/bladder ultrasound, and, potentially, computed tomography. Decision to pursue abdominal ultrasonography should be guided by clinical history, physical examination and laboratory findings suggestive of intra-abdominal or renal trauma. Laboratory evaluation may include complete blood count, coagulation profile, serum chemistry, and urinalysis. Most importantly, a high index of suspicion for renal injury is necessary when evaluating a neonate born after maternal trauma.

## 5. Summary Points

• Fetal kidney injury can occur after maternal motor vehicle accident and placental abruption, resulting in postnatal renal failure.
• The developing kidney has anatomic and developmental risk factors that increase susceptibility to traumatic injury.
• Evaluation of a neonate after suspected in utero trauma involves cranial ultrasound, abdominal ultrasound or focused renal/bladder ultrasound, and, potentially, computed tomography. Laboratory evaluation includes complete blood count, coagulation profile, serum chemistry, and urinalysis.

## Figures and Tables

**Table 1:** Neonatal Kidney Disease Improving Global Outcomes criteria for staging acute kidney injury [15].

Stage	Creatinine	Urine Output (mL/kg/Hour)	Duration (Hours)
0	No change in Cr or	≥0.5	
	Cr rise < 0.3 umol/L		
1	Cr rise > 0.3 umol/L in 48 h or	<0.5	6–12
	Cr rise > 1.5–1.9 times the lowest previous Cr		
2	Cr rise > 2.0–2.9 times the lowest previous Cr	<0.5	≥12
3	Cr rise > 3 times the lowest previous Cr or	<0.3 or anuria	≥12–24
	Cr > 2.5 umol/L or		
	Receipt of dialysis		

## Data Availability

The original contributions presented in this study are included in the article. Further inquiries can be directed to the corresponding author.

## Abbreviations

The following abbreviations are used in this manuscript:

AKI	Acute Kidney Injury
CT	Computed Tomography
DOL	Day of Life
MVA	Motor Vehicle Accident
NICU	Neonatal Intensive Care Unit
